# Immunogenicity of GX301 cancer vaccine: Four (telomerase peptides) are better than one

**DOI:** 10.1080/21645515.2015.1012032

**Published:** 2015-02-25

**Authors:** Daniela Fenoglio, Alessia Parodi, Rosa Lavieri, Francesca Kalli, Francesca Ferrera, Augusto Tagliamacco, Andrea Guastalla, Maria Giuseppina Lamperti, Mauro Giacomini, Gilberto Filaci

**Affiliations:** 1 Centre of Excellence for Biomedical Research; University of Genoa; Genoa, Italy; 2 Department of Internal Medicine; University of Genoa; Genoa, Italy; 3 IRCCS Azienda Ospedaliero Universitaria San Martino – IST – Istituto Nazionale per la Ricerca sul Cancro; Genoa, Italy; 4 Department of Informatics; Bioengineering, Robotics and System Engineering; University of Genoa, Genoa, Italy; 5 Mediolanum Farmaceutici s.p.a; Milan, Italy

**Keywords:** cancer vaccines, cytokine intracellular staining, elispot, GX301, immunogenicity, telomerase

## Abstract

Peptide_540–548_**,** peptide_611–626_, peptide_672–686_ and peptide_766–780_, which are derived from human telomerase, constitute the immunogenic component of the GX301 cancer vaccine. The relative immunogenicity of these peptides is unknown, thus it is unsure whether their combined use offers real advantages over single peptide stimulation. Hence, this study compared the number of specific immune responses and responders to each peptide, as well as to their mixture (meaning the co-presence of the 4 peptides in the same culture well), achieved after *ex vivo* stimulation of PBMC from 21, HLA-A2+ (n.11) or HLA-A2- (n.10), healthy donors. The study was performed on freshly collected PBMC (T0) and on PBMC stimulated for 10 d with single peptides or their mixture (T1). Peptide-specific immune responses were analyzed by Elispot and cytokine intracellular staining by flow cytometry. The results showed that each peptide induced specific immune responses in some subjects, with different panels of responders among the peptides. Moreover, the numbers of responses and responders to the single peptides or their mixture were comparable. Importantly, the overall number of responders to the 4 peptides was higher than to each single peptide, or to their mixture, both at T0 and T1. These data demonstrate the immunogenicity of each of the 4 GX301 telomerase peptides. Moreover, they show the advantage of multi-peptide over single peptide stimulation, providing a clear support to their combined administration in vaccination protocols. However, the data pose a warning against peptide administration as a mixture due to possible interference phenomena during antigen presentation processes.

## Abbreviations

CTL: cytotoxic T lymphocytes
PBMC: peripheral blood mononuclear cells
CIS: cytokine intracellular staining
IFNγ: interferon-gamma
PBS: phosphate buffered saline
FITC: fluorescein isothiocynate
mAb: monoclonal antibody

## Introduction

Telomerase is the reverse transcriptase that is responsible for the synthesis, elongation and stability of the telomeric regions of chromosomes.^1-4^ It is normally expressed by embryonic cells but not by adult somatic cells with few exceptions, and it is re-expressed by tumor cells since it is essential for tumor immortalization.^5-7^ Telomerase is immunogenic and telomerase-specific T cells have been identified both in healthy subjects and in cancer patients.^8,9^ In a previous study we observed that about 90% of cancer patients, with various histology and tumor stages, have circulating telomerase-specific cytotoxic T lymphocytes (CTL).^10^ All together these findings support the concept that telomerase may represent a universal tumor-associated antigen.^11^ Therefore, over the last decade several clinical trials have been carried out on cancer patients using telomerase as an immunogenic agent. When the rate of telomerase-specific immunological responses was evaluated as an outcome of telomerase immunization, conflicting results were observed among clinical trials.^12-19^ This raised concerns on the actual immunogenicity of telomerase, an issue further sustained by the fact that it is an endogenous antigen, as well as by the very low frequency of circulating telomerase-specific CD8+ T cells in cancer patients and by the inability of telomerase-specific CTL to kill tumor cells, as reported by some groups.^18,20,21^ Taken together, these concerns, impacted negatively on telomerase ranking in the prioritization list of tumor associated antigens that had been generated to identify the best candidates as immunogens for cancer vaccines.^22^

GX301 is a newly generated, multi-peptide, telomerase vaccine including 4 telomerase peptides (peptide_540-548_, peptide_611-626_, peptide_672-686_, peptide_766-780_) that are able to bind to both HLA class I and II molecules.^9,17,23,24,25^ In a recent phase I clinical trial the immunological and clinical effects of the GX301 vaccine were analyzed in a series of patients affected by stage IV prostate or renal cancers.^22^ The results of the study demonstrated the high immunogenicity of the GX301 vaccine, since all patients showed immune responses specific to the immunizing telomerase peptides associated with potential therapeutic efficacy.^23^ The high rate of telomerase-specific immunologic responses elicited by GX301 may depend on the fact that it is a multi-peptide vaccine. This likely circumvents the issues related both to the immunogenicity of telomerase and to HLA restriction of vaccinating peptides, also allowing activation of both CD4+ and CD8+ T cells subsets, which is required for optimal immune responses.^26^ Indeed, this study was designed to verify whether the immunogenicity (in terms of the number of specific immune responses and responders, as analyzed by measuring the induction of interferon-gamma (IFNγ)-secreting T cells by Elispot and by cytokine intracellular staining (CIS)) of the 4 GX301 peptides taken together is greater than that of each single peptide. It must be underlined that the analyses were performed in a cohort of healthy subjects, and not of cancer patients, in order to unveil the existence of spontaneous (not cancer-induced) immunoreactivity against GX301 telomerase peptides in the general population.

The results demonstrate that all subjects showed specific immune responses against al least one GX301 peptide and that the numbers of immune responses and responders induced by the 4 peptides taken together is greater than what was generated by each single peptide: hence, they validate the multi-peptide approach for telomerase immunization.

## Results

### Specific immune responses induced by each single peptide of the GX301 vaccine

Blood samples were collected from a cohort of 21 healthy donors and their peripheral blood mononuclear cells (PBMC) were *ex vivo* stimulated with 3 alternative concentrations (0.1, 1 and 10 μg/ml) of either single telomerase peptides (peptide_540–548_ named peptide A throughout the paper; peptide_611–626_ named peptide B throughout the paper; peptide_672–686_ named peptide C throughout the paper; peptide_766–780_ named peptide D throughout the paper) or their mixture (named ABCD throughout the paper), indicating with this term the co-presence of the 4 peptides in the same culture well. Analyses of immune responses were performed by 2 procedures, i.e., Elispot and CIS, at 2 time-points: T0 (on freshly isolated cells) and T1 (on short-term T cell lines generated after a 10-days incubation with or without the stimulatory peptide(s)). A schematic representation of the protocol design is provided in Supplementary **Figure 1**.

The issue concerning whether each peptide was able to induce T cell stimulation in some of the enrolled subjects was initially addressed. Supplementary **Tables 1 and 2** (relative to Elispot analyses) and Supplementary **Tables 3–6** (relative to CIS analyses) show that each peptide induced specific T cell responses in cells from more than one subject both at T0 and T1. Interestingly, the panels of responders to each peptide were specific for each single peptide and differed among peptides.

**Figure 1.**
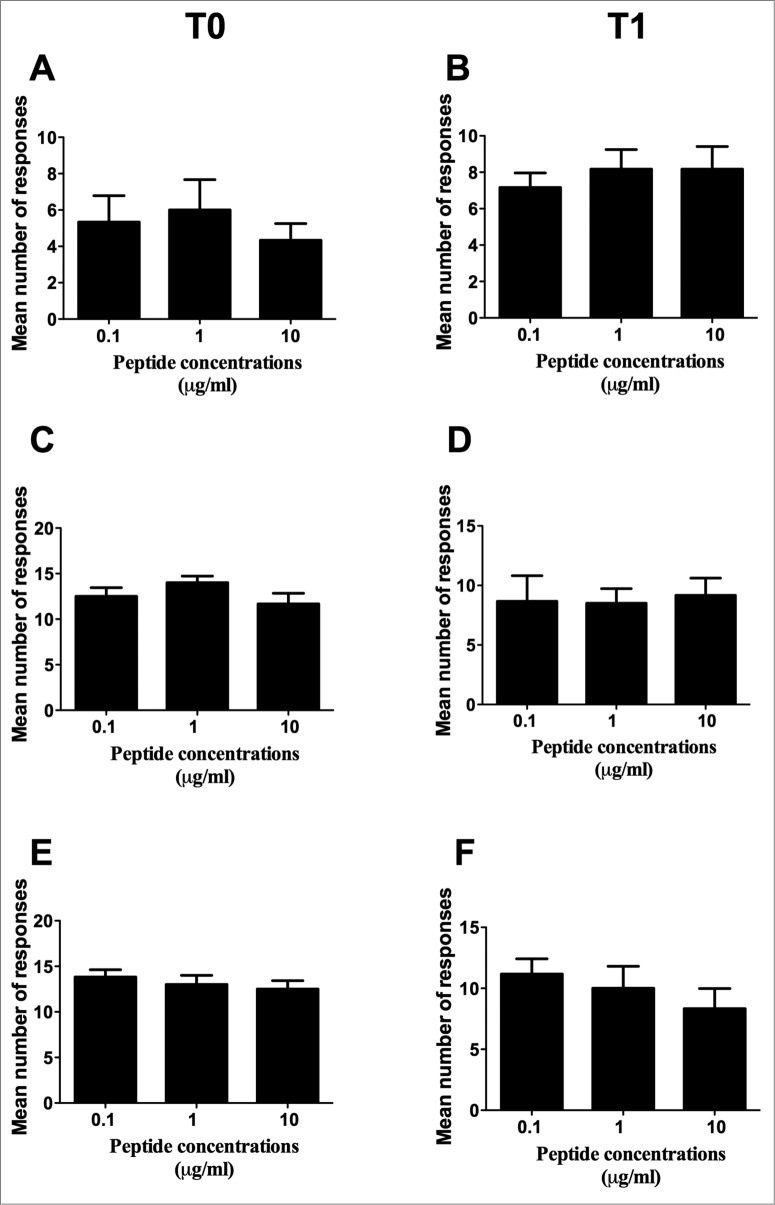
Comparison of the mean number of responses to the 4 peptides among the 3 different peptide concentrations. (**A** and **B**): Elispot analysis; (**C** to **F**): CIS analysis. (**C** and **D**): analyses on CD4+ T lymphocytes; (**E** and **F**): analyses on CD8+ T lymphocytes. **T0**: analyses performed on freshly isolated PBMC; **T1**: analyses performed on short-term peptide-specific T cell lines. Statistical analyses were performed by one-way ANOVA. Data are expressed as mean ± SD of positive responses (i.e., the count of positive responses developed by all subjects divided by the number of testing conditions (response to the 4 single peptides plus their mixture).

In order to verify whether peptide concentration had any relevance for the frequency of peptide-specific immune responses, the mean number of responses to the 4 peptides was compared among the 3 different concentrations. This analysis was performed both at T0 (**Figs. 1A, C, E**) and T1 (**Figs. 1B, D, F**) by either Elispot (**Figs. 1A and 1B**) or CIS (**Figs. 1C, D, E, F**), and showed no statistically significant differences. This finding therefore ruled out peptide concentration as a possible variable impacting on the frequency of peptide-specific immune responses, and allowed us to consider the total number of responses to the 3 peptide concentrations collectively in the following analyses and calculations.

### Comparative analysis of the total number of specific immune responses to the 4 peptides

**Figure 2.**
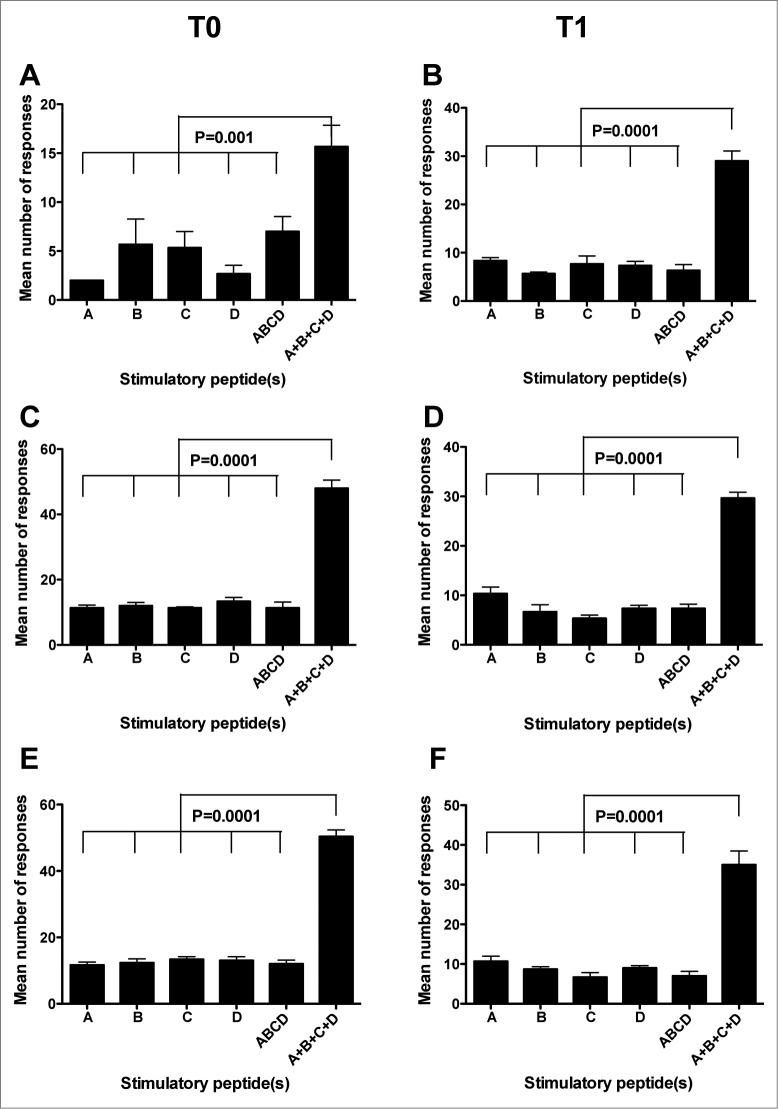
Comparison among the mean number of responses to the different stimulators. A, B, C, and D refer to the immune responses specifically achieved against each single peptide using peptide hTERT_540–548_, peptide hTERT_611–626_, peptide hTERT_672–686_ and peptide hTERT_766–780_, respectively, as the stimulator; ABCD refers to the immune responses specifically achieved against the mixture of the 4 peptides used as the stimulator; A+B+C+D refers to the sum of immune responses achieved against each single peptide. (**A** and **B**): Elispot analysis; (**C** to **F**): CIS analysis. (**C** and **D**): analyses on CD4+ T lymphocytes; (**E** and **F**): analyses on CD8+ T lymphocytes. **T0**: analyses performed on freshly isolated PBMC; **T1**: analyses performed on short-term peptide-specific T cell lines. Statistical analyses were performed by one-way ANOVA followed by Tukey's test. Data are expressed as mean ± SD of positive responses at the 3 different peptide concentrations (i.e., the number of positive responses developed by all subjects to each peptide and to their mixture divided by the number of tested peptide concentrations).

A first parameter we adopted to verify the existence of differences among the immunogenic potentials of the 4 peptides was the mean number of positive immune responses (detected by Elispot or CIS on PBMC from all the donors) that were specifically induced by each peptide or by their mixture at the 3 tested concentrations. **Figure 2** shows that no significant differences of this parameter were found by either Elispot or CIS analyses both at T0 and at T1 among single peptides as well as between single peptides and their mixture (indicated as ABCD). Instead, the mean of the sum of the specific immune responses to each single peptide (indicated as A + B + C + D) at the 3 concentrations was significantly higher than that of the numbers of immune responses to the single peptides or their mixture at the same concentrations (**Fig. 2**). This finding provides a formal demonstration of the immunogenic advantage (in terms of the number of elicited immune responses) offered by the multi-peptide composition of the GX301 telomerase vaccine.

**Figure 3.**
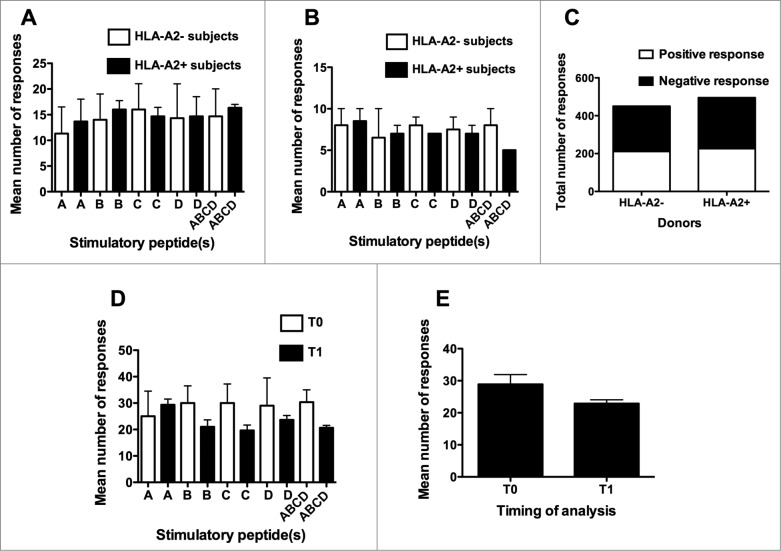
Comparison of the number of responses either between HLA-A2+ve and HLA-A2-ve donors or between T0 and T1 time-points of analysis. A, B, C, and D refer to the immune responses specifically achieved against each single peptide using peptide hTERT_540–548_, peptide hTERT_611–626_, peptide hTERT_672–686_ and peptide hTERT_766–780_, respectively, as the stimulator; ABCD refers to the immune responses specifically achieved against the mixture of the 4 peptides used as the stimulator. (**A**): analyses performed at T0; (**B**): analyses performed at T1; (**C**): Contingency analysis of frequencies of response in the 2 groups of donors; (**D**): analyses performed by comparing the number of responses to specific stimulators at T0 and T1; (**E**): analyses performed by comparing the total number of responses at T0 and T1. Statistical analyses were performed by one-way ANOVA (**A**, **B**, **D**, **E**) or Fisher's exact test (**C**). Data are expressed as: total mean ± SD of positive responses at the 3 different peptide concentrations (i.e., the number of all the positive responses to each peptide and to their mixture, detected both by Elispot and CIS, divided by the number of tested peptide concentrations) (**A, B, D** and **E**); total numbers of positive responses detected both by Elispot and CIS on PBMC from either HLA-A2+ve or HLA-A2-ve donors (**C**).

Interestingly, when the mean total (Elispot plus CIS) number of immune responses specific for each single peptide was taken into consideration, no significant differences were observed between HLA-A2- and HLA-A2+ donors (**Fig. 3**, panels A, B and C). Another relevant finding came from the comparison of the numbers of peptide-specific immune responses achieved at T0 and T1. Indeed, comparable mean numbers of immune responses were induced at the 2 time-points, either considering the immune response to the different peptides separately (**Fig. 3D**) or the mean number of immune responses to the 4 peptides and their mixture collectively (**Fig. 3E**).

With regard to the intensity of immune responses, in terms of the frequency of IFNγ positive spots (Elispot) or cells (CIS), no significant differences were observed among single peptides or their mixture (**Figs. S2 and S3**).

### Comparative analysis of the number of immunological responders to the 4 peptides

**Figure 4.**
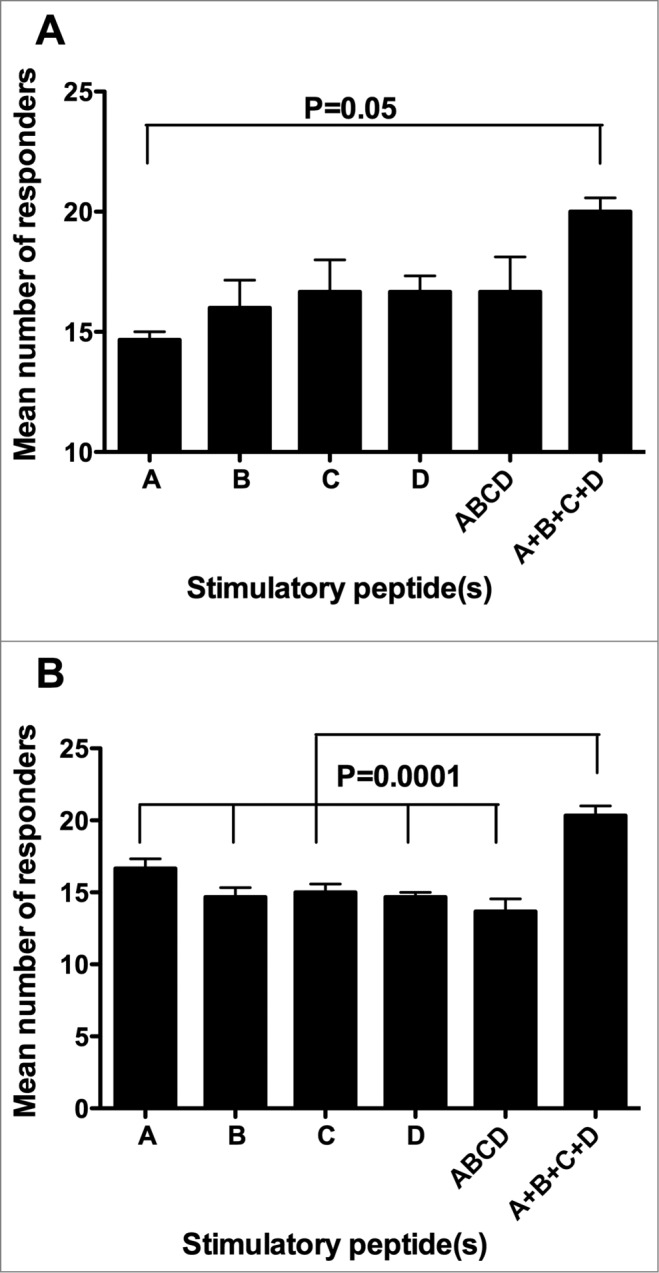
Comparison among the number of responders to the different stimulating conditions. A, B, C, and D refer to the donors showing specific immune responses against single peptides using peptide hTERT_540–548_, peptide hTERT_611–626_, peptide hTERT_672–686_ and peptide hTERT_766–780_, respectively, as the stimulator; ABCD refers to the donors showing specific immune responses against the mixture of the 4 peptides used as the stimulator; A + B + C + D refers to the sum of the number of responders to each single peptide at each tested concentration but counting subjects responsive to more than 1 peptide only once. (**A**): analyses performed at T0; (**B**): analyses performed at T1. Statistical analyses were performed by one-way ANOVA followed by Tukey's test. Data are expressed as total mean ± SD of positive responders at the 3 different peptide concentrations i.e., the number of all positive responders to each peptide and to the peptide mixture, detected either by Elispot or CIS, divided by the number of tested peptide concentrations).

Another parameter we took into consideration in order to evaluate the immunogenicity of the 4 telomerase peptides of the GX301 vaccine was the number of responders among our randomly selected cohort of healthy donors. The relevance of this parameter derives from the fact that, in the general population, the higher the number of individuals showing signs of immunization, the greater the probability of achieving a clinical response to the vaccine. **Tables 2 and 3** show that neither single peptides nor their mixture were able to induce specific immune response in all the tested individuals, regardless of the peptide concentration and timing of analysis. However, when we considered the overall number of responders to the 4 peptides, as calculated by summing the number of responders to each single peptide at each tested concentration, but counting subjects who were responsive to more than 1 peptide only once (in order to avoid replicate counts of responders), this number was always greater than the number of responders to either each single peptide or to the peptide mixture. Moreover, it was equal to the totality of the tested individuals (n. 21) at 3 experimental conditions (T0: 1 μg/ml peptide concentration; T1: 0.1 and 1 μg/ml peptide concentration). Accordingly, when the mean number of responders at the 3 peptide concentrations was considered cumulatively, the overall number of responders to the 4 peptides (A + B + C + D), calculated as above, proved to be higher than the number of responders to either each single peptide or to their mixture, reaching a statistically significant difference in the majority of comparisons (**Fig. 4**, panels A and B).

### Comparative analysis between the number of responses detected by Elispot or CIS

**Figure 5.**
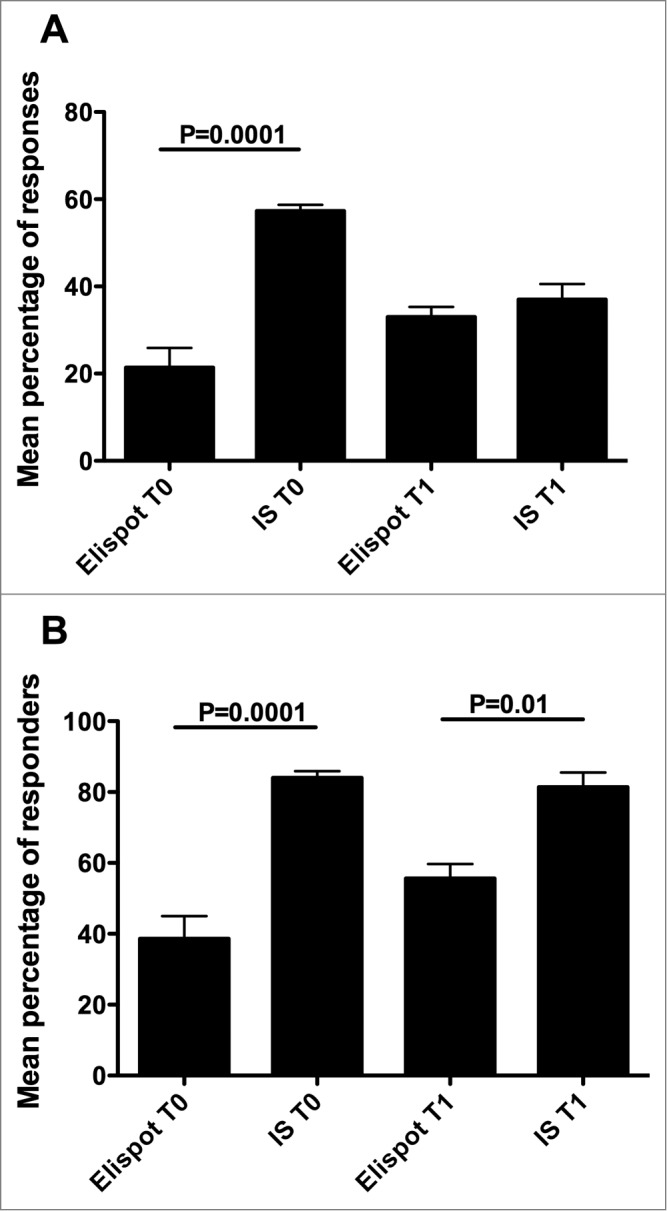
Comparison of the number of responses (**A**) or responders (**B**) at the 2 time-points between Elispot and CIS analyses. Statistical analyses were performed by one-way ANOVA followed by Tukey's test. Data are expressed as mean ± SD of percentages of responses (**A**) or responders (**B**) to each single peptide and to their mixture at the 3 different peptide concentrations.

This study was performed by applying 2 analytical procedures, Elispot and CIS, which are widely used for monitoring T cell responses to vaccinations and thus it could be important to comparatively verify the relative efficacy in detecting antigen-specific T cell responses. Here, a comparison of the total number of responses achieved by either procedure demonstrated that collectively considering the immune responses specific to each peptide and their mixture at the 3 peptide concentrations, the percentage of responses that were detected by CIS at T0 was significantly higher than what was detected by Elispot (Supplementary **Tables 7 and 8**, **Figs. 5A**). Similarly, the percentages of responders to each peptide and their mixture at the 3 peptide concentrations detected by CIS both at T0 and T1 was significantly higher than what was detected by Elispot (Supplementary **Tables 9 and 10**, **Fig. 5B**). Importantly, **Tables 4 and 5** highlight how some responders would have been missed if only one procedure had been applied.

### Tumor cell recognition by peptide-specific T cell lines

**Figure 6.**
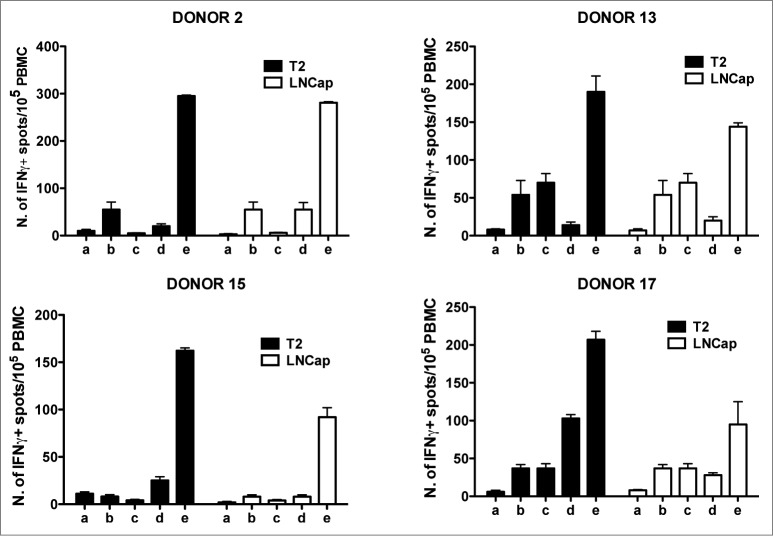
Elispot analysis of the reactivity of GX301 peptide-specific T cell lines from donors N. 2, 13, 15 and 17 against T2 and LNCap, telomerase-expressing tumor cell lines. Panels A, B, C and D refer to analyses performed with the T cell line against peptides C from donor N. 2, the T cell line against peptide B from donor N. 13, the T cell line against peptide A from donor N. 15, and the T cell line against peptide D from donor N. 17, respectively. Analyses were performed using T2 (black bars) or LNCap (open bars) tumor cell lines as target cells. a) tumor target cells alone; b) freshly purified autologous donor PBMC alone; c) peptide-specific T cell line alone; d) PBMC plus tumor target cells; e) peptide-specific T cell line plus autologous PBMC plus tumor target cells.

Data reported herein indicate that 100% of tested individuals showed immune reactivity against some of the telomerase peptides included in the GX301 vaccine. In order to verify whether these responses might have a protective value, peptide-specific T cell lines from different donors were tested for their ability to recognize tumor cell lines expressing telomerase. Thus, 2 tumor cell lines, T2 lymphoblastoid cells^27^ and LNCap prostate cancer cells,^28^ were preliminarily tested for telomerase expression and typed for HLA class I molecule expression. Supplementary **Figure 4** shows that both cell lines expressed telomerase; the HLA typing demonstrated that T2 cells were positive for HLA-A2, HLA-B51 and HLA-BW4 molecules, while LNCap cells expressed HLA-A1, HLA-A2, HLA-B8, HLA-B37, HLA-BW4 and HLA-BW6 molecules (not shown). At the same time, a panel of PBMC from HLA-A2+ and HLA-A2- donors were typed for HLA class I molecule expression. Among them, 2 HLA-A2+ (N. 13 and 15) and two HLA-A2- (N. 2 and 17) donors were selected since their haplotype partially matched that of the 2 tumor cell lines (Supplementary **Table 11**). Peptide-specific T cell lines were newly generated from each donor through short-term (10-days) culture of PBMC with each of the 4 telomerase peptides of the GX301 vaccine (at 10 μg/ml final concentration). At the end of this culture, each T cell line was tested by Elispot for its specific reactivity against the peptide that had been used for the short-term expansion. In these assays, the p66_460–480_ peptide derived from the HIV reverse transcriptase protein^29^ was used as an unrelated control peptide, in order to confirm the specificity of antigen recognition by the T cell lines. Based on these analyses, the T cell lines showing the highest peptide-specific responses (namely, the T cell line against peptide C for Donor N. 2, the T cell line against peptide B for Donor N. 13, the T cell line against peptide A for Donor N. 15, and the T cell line against peptide D for Donor N. 17) (**Table 6**) were selected. **Figure 6** shows that the 4 T cell lines selected from the different donors were all able to react specifically against T2 and LNCap tumor cell lines, while this was not the case for freshly purified PBMC. This result suggests that stimulation with GX301 peptides expands/activates an effector T cell subpopulation among low- or un-reactive PBMC that is able to recognize telomerase-expressing tumor cells.

**Table 1.** Subject's characteristics

Subject N.	Sex	Age	HLA-A2*
1	Female	56	−
2	Male	50	−
3	Female	32	+
4	Male	27	+
5	Female	40	−
6	Male	27	+
7	Male	59	+
8	Female	29	+
9	Female	28	+
10	Female	28	+
11	Female	44	+
12	Female	46	+
13	Male	40	+
14	Male	53	−
15	Male	43	+
16	Female	62	−
17	Female	49	−
18	Male	29	−
19	Male	45	−
20	Female	38	−
21	Female	37	−

*+: HLA-A2 positive; -: HLA-A2 negative.

**Table 2.** Numbers of responders to the different peptide stimulations at T0

Peptide concentrations	A*	B	C	D	ABCD**	A + B + C + D***
0.1 μg/ml	15	16	18	18	19	20
1 μg/ml	14	18	18	15	17	21
10 μg/ml	15	14	14	16	14	19

*: A, B, C, and D refer to the numbers of donors showing specific immune responses against each single peptide using peptide hTERT_540–548_, peptide hTERT_611–626_, peptide hTERT_672–686_ and peptide hTERT_766–780_, respectively, as the stimulator; **: ABCD refers to the numbers of donors showing specific immune responses against the mixture of the 4 peptides used as the stimulator; ***: A+B+C+D refers to the sum of the number of responders to each single peptide at each tested concentration but counting subjects who were responsive to more than 1 peptide only once.

**Table 3.** Numbers of responders to the different peptide stimulations at T1

Peptide concentrations	A*	B	C	D	ABCD**	A+B+C+D***
0.1 μg/ml	16	14	14	14	15	20
1 μg/ml	18	15	15	15	14	21
10 μg/ml	16	16	16	14	12	20

*: A, B, C, and D refer to the numbers of donors showing specific immune responses against each single peptide using peptide hTERT_540–548_, peptide hTERT_611–626_, peptide hTERT_672–686_ and peptide hTERT_766–780_, respectively, as the stimulator; **: ABCD refers to the numbers of donors showing specific immune responses against the mixture of the 4 peptides used as the stimulator; ***: A+B+C+D refers to the sum of the number of responders to each single peptide at each tested concentration but counting subjects who were responsive to more than 1 peptide only once.

**Table 4.** Distribution among subjects of positive responses to the different peptide stimulations detected by either Elispot or CIS at T0

	Stimulatory peptide(s)									
Subjects (n.)	A*		B		C		D		ABCD**	
	Elispot	CIS	Elispot	CIS	Elispot	CIS	Elispot	CIS	Elispot	CIS
1	−***	−	−	+	−	+	−	+	−	−
2	−	+	+	+	+	+	−	+	+	+
3	−	+	+	+	−	+	−	+	+	+
4	−	+	+	+	+	−	−	+	+	−
5	−	+	−	+	−	+	−	−	−	+
6	−	+	−	+	−	−	−	+	−	+
7	−	+	−	+	−	+	−	+	−	+
8	+	+	+	+	+	+	+	+	+	+
9	−	−	+	+	+	−	−	−	+	−
10	−	−	+	−	+	−	+	−	+	−
11	+	+	+	+	+	+	+	+	+	+
12	−	+	−	+	−	+	−	+	−	+
13	+	+	+	+	+	+	+	+	+	+
14	−	+	−	−	−	+	−	+	−	+
15	−	+	+	+	+	+	+	+	+	+
16	+	+	+	+	+	+	+	+	+	+
17	−	+	−	+	−	+	−	+	−	+
18	−	+	−	+	+	+	−	+	−	+
19	−	+	−	+	−	+	−	+	−	+
20	−	+	+	+	−	+	−	+	−	+
21	−	+	−	+	−	+	−	+	−	+

*A, B, C, and D refer to the numbers of immune responses specifically achieved against each single peptide using peptide hTERT_540–548_, peptide hTERT_611–626_, peptide hTERT_672–686_ and peptide hTERT_766–780_, respectively, as the stimulator; **ABCD refers to the immune responses specifically achieved against the mixture of the 4 peptides used as the stimulator; ***+: positive response; -: negative response.

**Table 5.** Distribution among subjects of positive responses to the different peptide stimulations detected by either Elispot or CIS at T1

	Stimulatory peptide(s)									
Subjects (n.)	A*		B		C		D		ABCD**	
	Elispot	CIS	Elispot	CIS	Elispot	CIS	Elispot	CIS	Elispot	CIS
1	−***	+	−	+	−	+	−	+	−	+
2	+	+	−	+	+	−	−	+	+	+
3	+	+	+	+	+	+	+	+	+	−
4	+	+	+	+	+	+	+	+	+	+
5	+	+	+	+	+	+	+	+	+	+
6	+	+	−	+	−	+	+	+	−	+
7	−	+	+	+	+	−	−	−	+	−
8	−	+	−	+	−	−	−	+	−	−
9	−	+	−	+	−	+	−	+	−	+
10	+	+	−	+	+	−	−	+	−	−
11	+	+	+	−	+	+	+	+	+	+
12	+	+	+	−	+	−	+	−	+	−
13	−	+	−	+	−	+	−	+	−	+
14	+	+	−	+	+	+	+	−	+	+
15	+	−	+	−	+	−	+	−	−	−
16	−	+	−	+	+	+	+	+	−	+
17	−	+	−	+	−	+	−	+	−	+
18	+	+	−	+	+	+	+	+	−	+
19	−	+	−	+	−	+	−	+	+	+
20	+	+	+	+	+	+	+	+	+	+
21	+	+	+	+	+	+	+	+	+	+

*A, B, C, and D refer to the numbers of immune responses specifically achieved against each single peptide using peptide hTERT_540–548_, peptide hTERT_611–626_, peptide hTERT_672–686_ and peptide hTERT_766–780_, respectively, as the stimulator; **ABCD refers to the immune responses specifically achieved against the mixture of the 4 peptides used as the stimulator; ***+: positive response; -: negative response.

**Table 6.** Elispot analysis of antigen-specificity of T cell lines from donors N. 2, 13, 15 and 17

		T cell line reactivity against:				
Donor N.	Peptide used for T cell line expansion	Unrelated peptide	Peptide A	Peptide B	Peptide C	Peptide D
2	A	9*	7			
	B	8		12		
	C	9			23	
	D	8				16
13	A	10	13			
	B	11		25		
	C	10			8	
	D	12				21
15	A	14	23			
	B	15		17		
	C	14			17	
	D	13				15
17	A	24	28			
	B	25		39		
	C	28			121	
	D	27				151

* Data are expressed as the number of IFNγ+ spots/10^5^ PBMC. A T cell line was considered peptide-specific when the number of spots elicited by stimulation with the GX301 telomerase peptide used for *ex vivo* short-term expansion was ≥30% of the background spot number (represented by the reactivity against the p66_460–480_ peptide derived from the HIV reverse transcriptase protein).

## Discussion

The results of this study indicate that: a) each GX301 peptide showed the ability to induce specific immune responses in some, but not all, subjects, although the panel of responders to each single peptide differed among peptides; b) both the numbers of responses and the numbers of responders to each single peptide, or to their mixture, were comparable among the different peptides; c) the total number of responses or responders to the 4 peptides was higher than to single peptides, or to their mixture, both at T0 and T1.

In the case of endogenous antigens, immunoreactivity against tumor associated antigens may be limited by the induction of immune tolerance, i.e., through intrathymic deletion.^30^ With regard to telomerase, although previous studies have reported telomerase-specific T lymphocytes in healthy individuals and in cancer patients, a systematic analysis on its immunogenicity in the general population is lacking.^8,9,20^ Nonetheless, the GX301 cancer vaccine proved to be highly immunogenic in a series of prostate and renal cancer patients in advanced stages of disease.^23^ The reasons for such high immunogenicity of the GX301 vaccine could reside either in a high level of immunogenicity intrinsic for one/some particular telomerase peptide(s) included in the vaccine, or in the fact that it is a multi-peptide vaccine, in which the 4 different peptides may mediate additive immunogenic effects. Discerning among these different mechanisms is clinically relevant since ethics and good clinical practice require that only truly effective agents must be administered to patients. Hence, demonstration of poor or absent immunogenic efficacy by one or more of the GX301 peptides would warrant the exclusion of this (these) agent(s) from the vaccine itself. Therefore, an analysis on the specific immunogenicity of each peptide and of their association is mandatory in order to fulfil the clinical regulatory requirements. Thus, this study was designed to analyze the immunogenicity of the 4 telomerase peptides included in the GX301 vaccine when they are used as immune stimulators (separately or in a mixture) for the PBMC taken from a cohort of healthy donors. In particular, the aim of the study was to provide an answer to the question related to which advantages, in terms of immunoreactivity, may be offered by the use of more than one telomerase peptide as an immunogen with respect to the use of single peptides. Indeed, the use of multi-peptide vaccines has been suggested as a way to increase immunization efficiency, in particular in the case of telomerase.^31^ However, a formal demonstration of the increased immunogenicity provided by the association of multiple telomerase peptides is lacking.

It is important to underline that the study was performed on PBMC from a cohort of healthy subjects, who had not been pre-vaccinated with telomerase. This explains the relatively low frequency of the immune responses we observed, which is typical of primary immune reactions. Since we were worried that the spontaneous immunoreactivity against the peptides would be so low as to be undetectable, the analyses were performed not only on freshly isolated PBMC but also on PBMC shortly *ex vivo* stimulated with the peptides (in order to slightly select/expands peptide-specific T cell lines). However, comparable findings were achieved at the 2 time-points, thus excluding the possibility that the short-term *ex vivo* stimulation could have artificially altered the results. Interestingly, each single peptide induced immune responses in different individuals, proving to be immunogenic in at least part of the general population. Moreover, the analyses performed by CIS allowed us to demonstrate that each of the 4 peptides could induce both CD4+ and CD8+ T cell responses. This is not surprising since 3 out of the 4 telomerase peptides included in the GX301 vaccine are known to be promiscuous peptides that are able to bind to both HLA class I and II molecules.^24,25^ Indeed, promiscuity of peptide binding to HLA molecules and T cell receptor degeneracy are well known mechanisms that allow the immune system to mature by developing a wide T cell repertoire that can recognize a huge array of antigen specificities.^32-34^ Therefore, our results indicate that the epitopes that are present in the GX301 peptides may be commonly expressed in the thymus where they select both CD4+ and CD8+ specific T cell clones. Accordingly, 100% of the subjects in our study showed an immune response to at least one peptide, regardless of the expression of the HLA-A2 molecule. Importantly, the T cell lines specifically responding to a stimulating GX301 peptide were also able to efficiently react against telomerase-expressing tumor cell lines, thus demonstrating their potential anti-tumor activity. These observations suggest that immunoreactivity against telomerase is a constitutive feature that is widespread among the general population, and that the haplotype coverage offered by the 4 peptides is broad enough to cover a large amount of haplotype specificities. On the basis of these observations, it would be of interest to carry out a future study to systematically analyze the haplotype restriction of each GX301 peptide using a wide panel of antigen presenting cells expressing different HLA alleles, in order to obtain data that would allow to make an approximate calculation of the probability of being a responder to the GX301 vaccine.

Another relevant result of the study is the demonstration that the mixture of the 4 telomerase peptides did not offer any greater immunogenic advantage with respect to the stimulation with single peptides. In fact, the number of specific immune responses achieved by the mixture of the peptides was generally comparable to what was obtained by the single peptides. It can be hypothesized that the occurrence of reciprocal interference among peptides, during their processing and loading into HLA molecules by the antigen presenting cell machinery, is responsible for the results.^35^ This finding has an immediate translational relevance since it suggests the need to inject these telomerase peptides into separate areas of the skin, when administering the vaccine in order to avoid the occurrence of local reciprocal interference.

Importantly, when we calculated the overall number of responders to the 4 peptides by summing the number of all responsive individuals but counting subjects responsive to more than 1 peptide only once (in order to avoid replicate counts of responders), this number was always higher than the number of responders to each single peptide or to their mixture. Despite the limitations of an *ex vivo* study, this observation provides a formal support to the concept that the multi-peptide composition of the GX301 vaccine may offer significant advantages in terms of immunogenicity with respect to the use of single peptides as immunogens. Accordingly, this study, in which 100% of individuals demonstrated immunoreactivity against the telomerase peptides, replicated the results that were observed in the recent clinical trial performed with the GX301 vaccine.^23^ Hence, it strongly suggests that although human telomerase is a self antigen, it does not undergo to relevant tolerogenic phenomena that can impede the onset of specific immune responses.

A final consideration concerns a technical aspect: the efficacy and appropriateness of Elispot and CIS in detecting the onset of peptide-specific immune responses after vaccination. This is an important issue since internationally shared guidelines driving the choice among immunological tests for the follow-up of immunotherapies are still lacking.^36^ In particular, it is unclear whether Elispot and CIS should be used as alternative procedures or in association in order to obtain more reliable results. Our data provide insights on this issue. In fact, the number of immune responses, as well as of responders, that were identified by CIS was higher than what was detected by Elispot. This suggests that, in our experimental setting, CIS showed a greater sensitivity than Elispot in detecting peptide-specific immune responses. However, some responses/responders would have been missed had Elispot analysis not been performed. Hence, these data suggests that although both Elispot and CIS focus on the detection of antigen-specific cytokine production by T lymphocytes, they should be considered as complementary rather than alternative analyses. Indeed, what remains to be clarified by future studies is how predictive these immunological tests are of the clinical response and whether their predictive value could be enhanced by the association with a peptide-specific cytotoxic assay.

Collectively, this study demonstrates the constitutive and widespread presence of telomerase-specific T cells in a cohort of healthy Caucasian subjects. In particular, this immune responsiveness, which targets the 4 GX301 telomerase peptides, differs among individuals as far as the target peptide(s) is/are concerned. Indeed, the stimulation of a subject with the 4 peptides offers advantages, in terms of the number of specific immune responses and the likelihood of being a responder, with respect to the use of a single peptide as an immunogen, thus providing a rationale for the multi-peptide composition of the GX301 vaccine. Future studies are needed to analytically define the allelic HLA restriction pattern of GX301 peptides as well as to clarify the ability of Elispot and CIS to predict clinical responses associated with vaccine-specific immunological reactivity.

## Materials and Methods

### Ethics and subjects

The protocol was approved by the local Ethics Committee at the IRCCS – AOU San Martino – IST, Genoa, Italy. The study was performed in accordance with the Declaration of Helsinki and with Good Clinical Practice as defined by the International Conference on Harmonization. All subjects gave voluntary, written informed consent at the time of their definitive enrolment or during the screening period.

Twenty-one healthy individuals, all of Caucasian race, were enrolled. They were preliminarily screened for HLA-A2 haplotype expression: 11 subjects were found HLA-A2+ve while 10 subjects were HLA-A2-ve. The characteristics of the study population are described in **Table 1**.

### Peptides

The following telomerase peptides, constituting the antigenic part of the GX301 vaccine, were used as in vitro immunogens in the study:

peptide_540–548_ (named peptide A throughout the paper); peptide_611–626_ (named peptide B throughout the paper); peptide_672–686_ (named peptide C throughout the paper); peptide_766–780_ (named peptide D throughout the paper). The peptides were provided by Bachem AG.

The p66_460–480_ peptide derived from the HIV reverse transcriptase protein, which was used as negative control in some experiments, was a kind gift from Prof. Fabrizio Manca. ^29^

Single peptides were stored as lyophilized powder in 500 μg vials at −20°C. Each peptide vial was dissolved in 1 ml of sterile phosphate buffered saline (PBS) and used in the cultures at the final concentrations of 0.1, 1 and 10 μg/ml.

### Tumor cell lines

T2 lymphoblastoid cells (174 × CEM.T2, ATCC® CRL1992™) and LNCap prostate cancer cells (LNCap clone FGC, ATCC® CRL1740™) were both purchased from American Type Culture Collection (ATCC, Manassas, VA).

### Analysis of HLA-A2 expression

Analysis of HLA-A2 expression was performed by immunofluorescence. Briefly, 50 μl of peripheral blood were incubated with or without (negative control) the unconjugated anti-HLA-A2 BB7.2 monoclonal antibody (mAb) for 20 minutes at room temperature.^34^ Cells were washed once with PBS, and incubated with Goat anti Mouse-fluorescein isothiocyanate (FITC) labeled secondary antibody (Southern Biotech, Cat. N. 1030–02) for 30 minutes at room temperature. Red cells were lysed with BD FACS Lysing solution (Becton Dickinson, Cat. N. 349202 (BD) Biosciences) and analyzed by a BD FACS Canto II using the FACS Diva software (BD).

### Generation of short-term peptide-specific T cell lines

PBMC were isolated from heparinized blood using density-gradient centrifugation over Ficoll-Hypaque (Biochrom, Cat. N. L6115). Two × 10^6^ PBMC were cultured in RPMI added with 10% autologous plasma in the presence or not of single peptides or their mixture at 0.1, 1 and 10 μg/ml final concentration of each peptide. Human recombinant IL-7 cytokine (1000 U/ml) (rhIL-7, PeproTech, Cat. N. AF-200–07) and anti-human CD28 (BD, Cat. N. 555725) and anti-human CD49d mAbs (BD, Cat. N. 555501) at 1 μg/ml final concentration were added at the beginning of the cultures. After 3 days, human recombinant IL-2 (rhIL-2, PeproTech, Cat. N. 200–02) at 30 U/ml was added to the cultures. After 10 d of culture, cells were harvested and re-stimulated overnight with single peptides or their mixture at 0.1, 1 and 10 μg/ml final concentration of each peptide (as specified below) before analyzing them for the frequency of peptide-specific IFNγ-producing T cells by both Elispot and CIS.

### Elispot analyses

Elispot analyses were performed in order to detect T cell reactivity against either the GX301 peptides or tumor cell lines.

With regard to our first aim (i.e., reactivity against peptides), analyses were performed on freshly isolated PBMC (T0) and on short-term peptide-specific T cell lines (T1) using the Human IFNγ ELISPOT Kit according to the manufacturer's instructions (BD, Cat. N. 5514849) and following the indications coming from international proficiency panels.^38^ Briefly, PBMC (2 × 10^5^ cells resuspended in RPMI added with 10% autologous plasma) or cells from short-term peptide-specific T cell lines (2 × 10^5^ cells resuspended in RPMI added with 10% autologous plasma) were incubated overnight in the presence of anti-human CD28 and anti-human CD49d mAbs (BD) (both at 1 μg/ml), as well as with one of the following stimulators: a) single peptides or their mixture at 0.1, 1 and 10 μg/ml final concentration of each peptide; b) phytohaemagglutinin (PHA-P, MPBIO, Cat. N. 151884) 1 μg/ml, as positive control, as described elsewhere;^39,40^ c) medium alone or medium added with the p66_460–480_ peptide^29^ derived from the HIV reverse transcriptase protein, as negative controls, as adopted in international proficiency panels.^38^

Regarding our second aim (i.e., reactivity against tumor cell lines), cells from short-term peptide-specific T cell lines (1 × 10^5^ cells resuspended in RPMI added with 10% autologous plasma) were incubated overnight in the presence of anti-human CD28 and anti-human CD49d mAbs (BD) (both at 1 μg/ml), as well as with autologous irradiated (3000 rad) PBMC (1 × 10^5^ cells/well) and T2 or LNCap tumor cells (5 × 10^4^/well). Cultures of T2 or LNCap cells (5 × 10^4^/well) alone, as well as of PBMC (1 × 10^5^ cells/well) alone or co-cultured with T2 or LNCap tumor cells (5 × 10^4^/well) served as negative controls.

At the end of incubation the spots assessing IFNγ production were counted by the Elispot Reader (Automated Elisa-Spot Assay Video Analysis Systems, AELVIS). The mean number of spots was calculated and net results (corrected for background signals detected in samples in medium alone) were expressed as the number of spots per 10^5^ cells. To distinguish between positive and negative immune responses, a cut-off value of ≥30% background number of spots was considered as positive.

### Analysis of peptide-specific T cell frequency by CIS

Analyses were performed on freshly isolated PBMC (T0) and on short term peptide-specific T cell lines (T1) following international guidelines.^41^ PBMC (1 × 10^6^ cells resuspended in RPMI added with 10% autologous plasma) or cells from short-term peptide-specific T cell lines (3 × 10^5^ cells resuspended in RPMI added with 10% autologous plasma) were plated in 96 flat-bottomed well plates (Orange Scientific, Cat. N. 5530200) and incubated overnight in the presence of anti-human CD28 and anti-human CD49d mAbs (BD), as well as with single peptides or their mixture, at 0.1, 1 and 10μg/ml final concentration of each peptide. Brefeldin (10 μg/ml, Sigma, Cat. N. B7651) was added to the cells for the last 3 hours of incubation. After washings, the samples were stained with fluorochrome-conjugated antibodies specific for surface markers and vitality dye (phycoerythrin-conjugated anti-human CD8, BD, Cat. N. 555367, and allophycocianin-conjugated anti-human CD3, BD, Cat. N. 555339), and Violet Live/Dead Fixable Dead Cell stain (Life Technologies, Cat. N. L34955), before fixing and permeabilizing the lymphocytes with the Cytofix/Cytoperm kit (BD, Cat. N. 554722) following the manufacturer's instructions. The cells were washed in Perm-Wash buffer (BD, Cat. N. 554723) and incubated with a FITC-conjugated anti-human IFNγ mAb (BD, Cat. N. 557718). Thereafter the samples were washed in Perm-Wash buffer, fixed with FACS Lysing solution (BD) and analyzed by a FACSCanto flow cytometer (BD) using the FACS Diva software (BD). In order to distinguish between positive and negative immune responses, a cut-off value of ≥0.1% background positive cells was considered as positive, as suggested for low frequency reactivities.^41^

### Analysis of telomerase expression

This analysis was performed on T2 and LNCap tumor cell lines as well as on PBMC from donor N. 1 for comparison. The quantitative detection of mRNA encoding for human telomerase catalytic subunit (hTERT) was performed by real time-PCR as follows: 48 μl of total RNA, isolated using the OMNIZOL RNA Isolation kit (EuroClone, Pero-Milan, IT), were treated with 6 U DNase I and reverse transcribed into cDNA using Oligo(dT) 20Primer and Superscript II Reverse Transcriptase (Invitrogen, Carlsbad, CA), followed by RNase H digestion. The reverse transcription was performed in the T100 Thermal Cycler (BioRad Laboratories, Hercules, CA 94547) under the following conditions: a single denaturation step at 94°C for 3 min followed by 35 cycles at 94°C for 1 min, at 64°C for 1 min and at 72°C for 2 min, followed by a final extension step at 72°C for 10 min. Quantitative Real Time PCR was performed using the LightCycler Nano thermocycler (Roche Diagnostics, Basel, Switzerland), the SYBR Green Master Mix (Roche Diagnostics, Basel, Switzerland) and gene specific primer pairs specific for either hTERT (FOR: 5′ TGA CAC CTC ACC TCA CCC AC-3′, REV: 5′-CAC TGT CTT CCG CAA GTT CAC-3′) or for glyceraldehyde-3-phosphate dehydrogenase (GAPDH) (FOR: 5′-GGC ATC CTG GGC TAC ACT GA-3′, REV: 5′-TGG TGG TCC AGG GGT CTT-3′) (TIB Molbiol, Genoa, Italy) mRNA. Reaction products were separated on a 1.2% agarose gel in TAE (40 mM Tris-acetate, 1 mM EDTA) containing SYBR Safe DNA gel stain (Invitrogen,Carlsbad, CA). hTERT PCR products were validated by sequence analysis. hTERT cDNA quantitation was normalized to GAPDH expression using the 2-ΔΔCT method

### HLA class I typing

Genomic DNA was extracted from whole blood samples using the QIAmp DNA Mini Kit (QIAGEN Gmbh, Hilden, Germany). Recipient low-resolution HLA-A*, HLA-B* typing was performed using polymerase chain reaction-sequence-specific primers (PCR-SSP) contained in the HLA-A and HLA-B SSP KIT (BIO-RAD, Dreieich, Germany). Then PCR products were then run by electrophoresis on agarose gel (2%) stained with VistraGreen TM (Amersham, Braunschweig, Germany) using 10 μL of the reaction mixture.

### Statistical analyses

Raw data were organized within an ad hoc developed web based database that retained the features of the performed experiments. This tool was able to efficiently prepare tables from data to be presented to commercial statistical packages.^42^

In all analyses, variables with identical sample size were compared. The differences among mean numbers of responses, or of responders, as well as the differences among frequencies of peptide-specific T lymphocytes were analyzed by one-way ANOVA. For cases in which the null hypothesis was rejected by ANOVA, the significantly different values were singled out by Tukey's test.

The differences in the numbers of responses between HLA-A2+ve and HLA-A2-ve subjects were analyzed by Fisher's exact test.

Differences were considered statistically significant when P < 0.05. All statistical analyses were performed using the GraphPad Prism 4.0 Software, Inc., La Jolla, CA, USA.
