# Supplementary material for: Immunogenicity of GX301 cancer vaccine: Four (telomerase peptides) are better than one
Source: Hum Vaccin Immunother. 2015 Feb 25;11(4):838–50. doi: 10.1080/21645515.2015.1012032 (PMC4514186; doi:10.1080/21645515.2015.1012032)
Supplement: Supplemental_Material.zip [file khvi-11-04-1012032-s001.zip › Supplementary Figures second revision.docx]

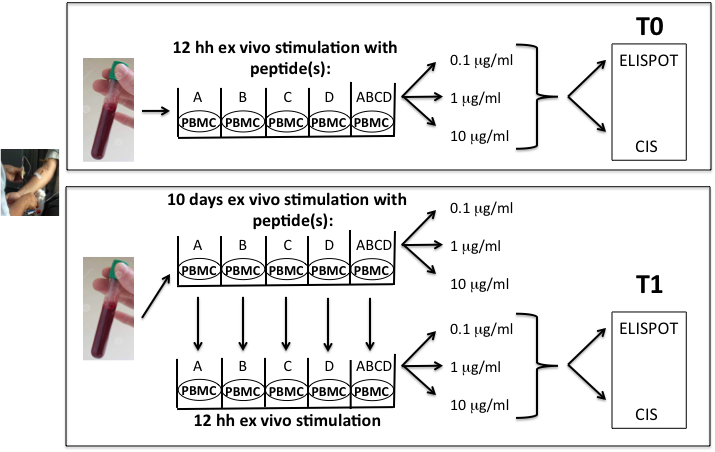


**Supplementary Figure 1**. Summary of study design. A, B, C, and D refer to peptide hTERT_540-548_, peptide hTERT_611-626_, peptide hTERT_672-686_ and peptide hTERT_766-780_, respectively; ABCD refers to the mixture of the four peptides used as the stimulator. PBMC: peripheral blood mononuclear cells.


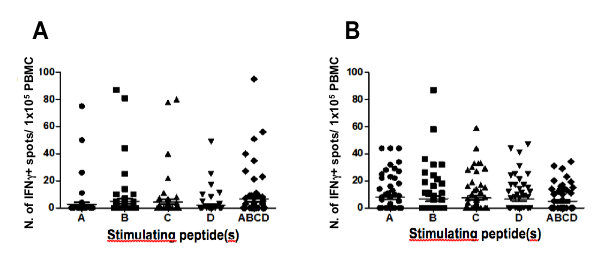
**Supplementary Figure 2.**  Frequencies of IFNγ+ spots induced by peptide-specific T lymphocytes analyzed by Elispot at T0 and T1. A, B, C, and D refer to the immune responses specifically achieved against each single peptide using, as stimulator, peptide hTERT_540-548_, peptide hTERT_611-626_, peptide hTERT_672-686_ and peptide hTERT_766-780_, respectively; ABCD refers to the immune responses specifically achieved against the mixture of the four peptides used as stimulator. **Panel A**: analyses performed at T0; **Panel B**: analyses performed at T1. Statistical analyses were performed by one-way ANOVA: no statistically significant differences were observed.

**Supplementary Figure 3.**  Frequencies of peptide-specific CD4+ (**Panels A** and **C**) and CD8+ (**Panels B** and **D**) T lymphocytes analyzed by CIS at T0 and T1. A, B, C, and D refer to the immune responses specifically achieved against each single peptide using, as stimulator, peptide hTERT_540-548_, peptide hTERT_611-626_, peptide hTERT_672-686_ and peptide hTERT_766-780_, respectively; ABCD refers to the immune responses specifically achieved against the mixture of the four peptides used as stimulator. **Panel A** and **B**: analyses performed at T0; **Panel C** and **D**: analyses performed at T1. Statistical analyses were performed by one way ANOVA: no statistically significant differences were observed.

**Supplementary Figure 4.** Quantitation by real time PCR of hTERT mRNA present in T2 and LnCap tumor cell lines as well as in PBMC from donor N. 1 for comparison. Data are expressed as relative hTERT mRNA expression compared to housekeeping GAPDH gene expression using the 2-∆∆CT method. The assay was performed in duplicate.
