# Supplementary material for: Immunogenicity of GX301 cancer vaccine: Four (telomerase peptides) are better than one
Source: Hum Vaccin Immunother. 2015 Feb 25;11(4):838–50. doi: 10.1080/21645515.2015.1012032 (PMC4514186; doi:10.1080/21645515.2015.1012032)
Supplement: Supplemental_Material.zip [file khvi-11-04-1012032-s001.zip › Supplementary Tables second revision.doc]

| **Supplementary Table 1. Number of IFNγ+ spots upon PBMC peptide stimulation at T0 analyzed by ELISPOT.** | | | | | | | | | | | | | | | |
| --- | --- | --- | --- | --- | --- | --- | --- | --- | --- | --- | --- | --- | --- | --- | --- |
| **Subject n.*** | **Stimulatory peptide(s)** | | | | | | | | | | | | | | |
|  | **A**  **0.1μg/ml** | **A**  **1μg/ml** | **A**  **10μg/ml** | **B**  **0.1μg/ml** | **B**  **1μg/ml** | **B**  **10μg/ml** | **C**  **0.1μg/ml** | **C**  **1μg/ml** | **C**  **10μg/ml** | **D**  **0.1μg/ml** | **D**  **1μg/ml** | **D**  **10μg/ml** | **ABCD 0.1μg/ml** | **ABCD 1μg/ml** | **ABCD 10μg/ml** |
| 2 | 0 | 0 | 0 | 0 | 2 | 0 | 4 | 0 | 0 | 0 | 0 | 0 | 4 | 0 | 4 |
| 3 | 0 | 0 | 0 | 0 | 10 | 0 | 0 | 0 | 0 | 0 | 0 | 0 | 9 | 0 | 0 |
| 4 | 0 | 0 | 0 | 0 | 14 | 7 | 6 | 0 | 0 | 0 | 0 | 0 | 21 | 27 | 23 |
| 8 | 0 | 0 | 11 | 0 | 44 | 25 | 22 | 8 | 0 | 17 | 25 | 0 | 35 | 8 | 40 |
| 9 | 0 | 0 | 0 | 0 | 10 | 0 | 0 | 7 | 0 | 0 | 0 | 0 | 9 | 0 | 0 |
| 10 | 0 | 0 | 0 | 0 | 6 | 4 | 6 | 6 | 0 | 5 | 0 | 0 | 9 | 11 | 0 |
| 11 | 2 | 0 | 0 | 0 | 2 | 5 | 1 | 0 | 2 | 3 | 0 | 0 | 3 | 2 | 3 |
| 13 | 75 | 50 | 26 | 0 | 81 | 87 | 78 | 80 | 40 | 10 | 0 | 49 | 95 | 51 | 56 |
| 15 | 0 | 0 | 0 | 0 | 4 | 0 | 0 | 4 | 0 | 0 | 0 | 8 | 7 | 0 | 0 |
| 16 | 0 | 4 | 0 | 0 | 6 | 5 | 7 | 4 | 0 | 0 | 0 | 11 | 7 | 0 | 5 |
| 18 | 0 | 0 | 0 | 0 | 0 | 0 | 0 | 11 | 0 | 0 | 0 | 0 | 0 | 0 | 0 |
| 20 | 0 | 0 | 0 | 5 | 0 | 0 | 0 | 0 | 0 | 0 | 0 | 0 | 0 | 0 | 0 |

*Only subjects showing at least one positive response are included. Data are expressed as the number of IFNγ+ spots/10^5^ PBMC.

| **Supplementary Table 2. Number of IFNγ+ spots upon PBMC peptide stimulation at T1 analyzed by ELISPOT.** | | | | | | | | | | | | | | | |
| --- | --- | --- | --- | --- | --- | --- | --- | --- | --- | --- | --- | --- | --- | --- | --- |
| **Subject n.*** | **Stimulatory peptide(s)** | | | | | | | | | | | | | | |
|  | **A**  **0.1μg/ml** | **A**  **1μg/ml** | **A**  **10μg/ml** | **B**  **0.1μg/ml** | **B**  **1μg/ml** | **B**  **10μg/ml** | **C**  **0.1μg/ml** | **C**  **1μg/ml** | **C**  **10μg/ml** | **D**  **0.1μg/ml** | **D**  **1μg/ml** | **D**  **10μg/ml** | **ABCD 0.1μg/ml** | **ABCD 1μg/ml** | **ABCD 10μg/ml** |
| 2 | 0 | 10 | 0 | 0 | 0 | 0 | 0 | 0 | 4 | 0 | 0 | 0 | 5 | 12 | 0 |
| 3 | 32 | 16 | 22 | 11 | 0 | 0 | 0 | 28 | 0 | 41 | 17 | 17 | 31 | 20 | 19 |
| 4 | 28 | 8 | 22 | 6 | 0 | 11 | 29 | 0 | 8 | 8 | 9 | 7 | 0 | 10 | 0 |
| 5 | 5 | 0 | 9 | 11 | 19 | 0 | 0 | 7 | 6 | 0 | 25 | 17 | 13 | 14 | 0 |
| 6 | 44 | 27 | 0 | 0 | 0 | 0 | 0 | 0 | 0 | 12 | 31 | 0 | 0 | 0 | 0 |
| 7 | 0 | 0 | 0 | 0 | 9 | 0 | 0 | 13 | 0 | 0 | 0 | 0 | 11 | 0 | 0 |
| 10 | 0 | 0 | 8 | 0 | 0 | 0 | 0 | 0 | 7 | 0 | 0 | 0 | 0 | 0 | 0 |
| 11 | 3 | 0 | 11 | 0 | 6 | 58 | 4 | 0 | 27 | 0 | 0 | 44 | 0 | 0 | 23 |
| 12 | 0 | 25 | 13 | 32 | 87 | 26 | 10 | 33 | 59 | 0 | 21 | 10 | 17 | 34 | 0 |
| 14 | 0 | 0 | 44 | 0 | 0 | 0 | 0 | 0 | 17 | 0 | 15 | 8 | 0 | 10 | 15 |
| 15 | 29 | 0 | 23 | 0 | 0 | 16 | 0 | 16 | 33 | 0 | 14 | 0 | 0 | 0 | 0 |
| 16 | 0 | 0 | 0 | 0 | 0 | 0 | 0 | 0 | 19 | 0 | 25 | 0 | 0 | 0 | 0 |
| 18 | 14 | 0 | 0 | 0 | 0 | 0 | 14 | 0 | 0 | 12 | 0 | 0 | 0 | 0 | 0 |
| 19 | 0 | 0 | 0 | 0 | 0 | 0 | 0 | 0 | 0 | 0 | 0 | 0 | 0 | 14 | 0 |
| 20 | 10 | 6 | 0 | 24 | 36 | 18 | 12 | 0 | 15 | 47 | 7 | 0 | 29 | 14 | 0 |
| 21 | 18 | 34 | 44 | 0 | 21 | 32 | 33 | 44 | 30 | 11 | 0 | 24 | 6 | 0 | 16 |

*Only subjects showing at least one positive response are included. Data are expressed as the number of IFNγ+ spots/10^5^ PBMC.

| **Supplementary Table 3. Frequencies of IFNγ secreting CD4+ T cells upon peptide stimulation at T0 analyzed by CIS.** | | | | | | | | | | | | | | | |
| --- | --- | --- | --- | --- | --- | --- | --- | --- | --- | --- | --- | --- | --- | --- | --- |
| **Subject n.*** | **Stimulatory peptide(s)** | | | | | | | | | | | | | | |
|  | **A**  **0.1μg/ml** | **A**  **1μg/ml** | **A**  **10μg/ml** | **B**  **0.1μg/ml** | **B**  **1μg/ml** | **B**  **10μg/ml** | **C**  **0.1μg/ml** | **C**  **1μg/ml** | **C**  **10μg/ml** | **D**  **0.1μg/ml** | **D**  **1μg/ml** | **D**  **10μg/ml** | **ABCD 0.1μg/ml** | **ABCD 1μg/ml** | **ABCD 10μg/ml** |
| 1 | 0 | 0 | 0 | 0.1 | 0 | 0 | 0 | 0 | 0 | 0 | 0 | 0.1 | 0 | 0 | 0 |
| 2 | 0 | 0 | 0 | 0.3 | 0 | 0.2 | 0.4 | 0 | 0.1 | 0.2 | 0.2 | 0.1 | 0.1 | 0.3 | 0.3 |
| 3 | 0 | 0.2 | 0.2 | 0 | 0.2 | 0.1 | 0.3 | 0.3 | 0.1 | 1.3 | 0.1 | 0.1 | 0 | 0.1 | 0.1 |
| 4 | 0.1 | 0 | 0 | 0 | 0 | 0 | 0 | 0 | 0 | 0 | 0 | 0 | 0 | 0 | 0 |
| 5 | 0.1 | 0.1 | 0 | 0.1 | 0 | 0 | 0.1 | 0.1 | 0 | 0 | 0 | 0 | 0.1 | 0 | 0 |
| 6 | 0.1 | 0.1 | 0.1 | 0 | 0.1 | 0 | 0 | 0 | 0 | 0.1 | 0.1 | 0 | 0.1 | 0 | 0 |
| 7 | 0.1 | 0.1 | 0.1 | 0.2 | 0.2 | 0 | 0.1 | 0.1 | 0.1 | 0.1 | 0.2 | 0.1 | 0.1 | 0.1 | 0.2 |
| 8 | 0 | 0.1 | 0 | 0.3 | 0.1 | 0.1 | 0.1 | 0 | 0 | 0 | 0.1 | 0.4 | 0.4 | 0.1 | 0 |
| 11 | 0 | 0 | 0.7 | 0 | 0.4 | 0 | 0 | 0.7 | 0 | 0 | 0 | 0.1 | 0 | 0.1 | 0 |
| 12 | 0.3 | 0.5 | 0.4 | 0.3 | 0.3 | 0.3 | 0.1 | 0.3 | 0.2 | 0.8 | 0.3 | 0.5 | 0.8 | 0.3 | 0 |
| 13 | 0 | 0 | 0 | 0 | 0.1 | 0 | 0 | 0 | 0 | 0 | 0.1 | 0.1 | 0 | 0.1 | 0 |
| 14 | 0.2 | 0.2 | 0 | 0 | 0 | 0 | 0 | 0.1 | 0 | 0 | 0.1 | 0 | 0 | 0 | 0.1 |
| 15 | 0.1 | 0.1 | 0.1 | 0.2 | 0.5 | 0.1 | 0.7 | 0.6 | 0.1 | 0.3 | 0.1 | 0.3 | 0.2 | 0.2 | 0 |
| 16 | 0 | 0 | 0.2 | 0.2 | 0 | 0.2 | 0 | 1.2 | 0.1 | 0.1 | 0.2 | 0 | 0 | 0.3 | 0 |
| 17 | 0.1 | 0.1 | 0 | 0.2 | 0.3 | 0.2 | 1.6 | 1.1 | 0.1 | 1 | 1.1 | 0.1 | 0.9 | 1.1 | 0.1 |
| 18 | 0 | 0.2 | 0 | 0.1 | 0.3 | 0 | 0.8 | 0 | 0.6 | 0 | 0.2 | 0.6 | 0.2 | 0.3 | 0.1 |
| 19 | 0.9 | 0.6 | 0.2 | 0.2 | 0.4 | 0.2 | 0 | 0.3 | 0.6 | 0.7 | 0.2 | 0.1 | 0.3 | 0.6 | 0.1 |
| 20 | 0.2 | 0.2 | 0.2 | 0.4 | 0.2 | 0.4 | 0.4 | 0.2 | 0.3 | 0.3 | 0.2 | 0.3 | 0.3 | 0.3 | 0.3 |
| 21 | 0.1 | 0.1 | 0.3 | 0.1 | 0.1 | 0.3 | 0.5 | 0.3 | 0.3 | 0.5 | 0.1 | 0.2 | 0.6 | 0.2 | 0 |

*Only subjects showing at least one positive response are included. Data are expressed as percentages of IFNγ secreting T cells/10^5^ PBMC.

| **Supplementary Table 4. Frequencies of IFNγ secreting CD8+ T cells upon peptide stimulation at T0 analyzed by CIS.** | | | | | | | | | | | | | | | |
| --- | --- | --- | --- | --- | --- | --- | --- | --- | --- | --- | --- | --- | --- | --- | --- |
| **Subject n.*** | **Stimulatory peptide(s)** | | | | | | | | | | | | | | |
|  | **A**  **0.1μg/ml** | **A**  **1μg/ml** | **A**  **10μg/ml** | **B**  **0.1μg/ml** | **B**  **1μg/ml** | **B**  **10μg/ml** | **C**  **0.1μg/ml** | **C**  **1μg/ml** | **C**  **10μg/ml** | **D**  **0.1μg/ml** | **D**  **1μg/ml** | **D**  **10μg/ml** | **ABCD 0.1μg/ml** | **ABCD 1μg/ml** | **ABCD 10μg/ml** |
| 1 | 0 | 0 | 0 | 0.3 | 0 | 0 | 0.1 | 0.1 | 0.4 | 0.2 | 0 | 0.3 | 0 | 0 | 0 |
| 2 | 0 | 0 | 0.2 | 0.3 | 0.2 | 0.2 | 0.4 | 0 | 0.1 | 0.2 | 0.3 | 0.2 | 0.4 | 0.3 | 0.6 |
| 3 | 0.2 | 0.9 | 0.9 | 0.7 | 0.5 | 0.6 | 1.6 | 0.7 | 0.2 | 2.4 | 0.6 | 0.5 | 0.1 | 0.5 | 0.6 |
| 4 | 0.1 | 0 | 0 | 0.1 | 0 | 0 | 0 | 0 | 0 | 0.1 | 0 | 0 | 0 | 0 | 0 |
| 5 | 0 | 0 | 0 | 0.1 | 0 | 0 | 0.1 | 0 | 0 | 0 | 0 | 0 | 0 | 0 | 0 |
| 6 | 0 | 0.1 | 0 | 0 | 0 | 0 | 0 | 0 | 0 | 0 | 0 | 0 | 0 | 0 | 0 |
| 7 | 0.1 | 0.2 | 0.3 | 0.5 | 0.4 | 0 | 0.2 | 0.4 | 0.3 | 0.3 | 0.5 | 0.5 | 0.2 | 0.3 | 0.3 |
| 8 | 0 | 0 | 0 | 0.2 | 0 | 0.1 | 0.1 | 0 | 0 | 0 | 0 | 0.5 | 0.2 | 0.1 | 0.1 |
| 9 | 0 | 0 | 0 | 0 | 0.2 | 0 | 0 | 0 | 0 | 0 | 0 | 0 | 0 | 0 | 0 |
| 11 | 0.1 | 0 | 0.3 | 0 | 0.2 | 0 | 0.1 | 0.3 | 0 | 0 | 0 | 0.1 | 0 | 0.1 | 0 |
| 12 | 0.4 | 0.7 | 0.6 | 0.3 | 0.5 | 0.4 | 0.1 | 0.3 | 0.2 | 1.6 | 0.4 | 1 | 1.1 | 0.3 | 0 |
| 13 | 0.2 | 0.5 | 0.5 | 0.6 | 0.5 | 0.8 | 0.3 | 0.3 | 0.6 | 0.4 | 0.7 | 0.3 | 0.5 | 1.1 | 0.4 |
| 14 | 0.1 | 0 | 0 | 0 | 0 | 0 | 0.1 | 0.1 | 0 | 0 | 0.1 | 0.1 | 0 | 0.1 | 0.1 |
| 15 | 0.3 | 0.3 | 1.2 | 1.2 | 1.7 | 1 | 1.9 | 1.7 | 1 | 1.1 | 0.7 | 0.4 | 0.4 | 0.5 | 0 |
| 16 | 0 | 0 | 1.5 | 0 | 0 | 0 | 0 | 0.5 | 0 | 0 | 0 | 0 | 0 | 0 | 0 |
| 17 | 0.5 | 0.3 | 0.1 | 0.3 | 0.5 | 0.2 | 1.1 | 0.7 | 0.1 | 0.7 | 0.7 | 0.1 | 0.7 | 0.7 | 0 |
| 18 | 0.2 | 0.9 | 1.7 | 1.6 | 0.8 | 0 | 1.6 | 0 | 0.5 | 0.1 | 0.2 | 0.7 | 0.3 | 0.3 | 0.1 |
| 19 | 2.1 | 1 | 0.3 | 0.7 | 1.3 | 0.3 | 0 | 0.9 | 0.9 | 3.1 | 1 | 0.4 | 1.4 | 2.6 | 0.7 |
| 20 | 0.1 | 0.2 | 0.2 | 0.5 | 0.3 | 0.6 | 0.6 | 0.3 | 0.4 | 0.3 | 0.2 | 0.4 | 0.7 | 0.7 | 0.8 |
| 21 | 0 | 0 | 0.1 | 0 | 0.1 | 0.2 | 0.6 | 0.3 | 0.3 | 0.3 | 0 | 0.3 | 2.5 | 0.8 | 0.1 |

*Only subjects showing at least one positive response are included. Data are expressed as percentages of IFNγ secreting T cells/10^5^ PBMC.

| **Supplementary Table 5. Frequencies of IFNγ secreting CD4+ T cells upon peptide stimulation at T1 analyzed by CIS.** | | | | | | | | | | | | | | | |
| --- | --- | --- | --- | --- | --- | --- | --- | --- | --- | --- | --- | --- | --- | --- | --- |
| **Subject n.*** | **Stimulatory peptide(s)** | | | | | | | | | | | | | | |
|  | **A**  **0.1μg/ml** | **A**  **1μg/ml** | **A**  **10μg/ml** | **B**  **0.1μg/ml** | **B**  **1μg/ml** | **B**  **10μg/ml** | **C**  **0.1μg/ml** | **C**  **1μg/ml** | **C**  **10μg/ml** | **D**  **0.1μg/ml** | **D**  **1μg/ml** | **D**  **10μg/ml** | **ABCD 0.1μg/ml** | **ABCD 1μg/ml** | **ABCD 10μg/ml** |
| 1 | 0.2 | 0.1 | 0 | 0 | 0 | 0 | 0.1 | 0.1 | 0.2 | 0 | 0 | 0.4 | 0 | 0.1 | 0.1 |
| 2 | 0 | 0 | 0.1 | 0.1 | 0 | 3.8 | 0 | 0 | 0 | 0.1 | 0 | 0 | 0 | 0.1 | 0 |
| 3 | 0.1 | 0.1 | 0.4 | 0 | 0.2 | 0 | 0 | 0.1 | 0.1 | 0 | 0 | 0.1 | 0 | 0 | 0 |
| 4 | 2.5 | 0.1 | 0.1 | 0 | 0.7 | 0.1 | 0 | 0 | 0 | 0 | 0 | 0.1 | 0.2 | 0 | 2.2 |
| 5 | 1 | 0 | 0.1 | 0 | 0 | 0.1 | 0 | 0 | 0 | 0.1 | 0.3 | 0 | 2.5 | 0.1 | 0 |
| 6 | 0 | 0 | 0 | 0 | 0 | 0.3 | 0 | 0 | 0 | 0 | 0 | 0.1 | 1.5 | 0.2 | 0 |
| 7 | 0 | 0.2 | 0.2 | 0.1 | 0 | 0 | 0 | 0 | 0 | 0 | 0 | 0 | 0 | 0 | 0 |
| 9 | 0.3 | 0 | 0 | 0 | 0.2 | 0 | 0 | 0 | 0 | 0.2 | 0 | 0 | 0.2 | 0 | 0.1 |
| 10 | 0 | 1 | 0 | 0 | 0 | 1.6 | 0 | 0 | 0 | 0 | 0.8 | 0.3 | 0 | 0 | 0 |
| 11 | 1.1 | 0 | 0 | 0 | 0 | 0 | 0 | 0 | 0.1 | 0 | 0 | 0 | 0 | 0 | 0 |
| 12 | 0.7 | 0 | 0 | 0 | 0 | 0 | 0 | 0 | 0 | 0 | 0 | 0 | 0 | 0 | 0 |
| 13 | 0 | 0.1 | 0 | 0 | 0.1 | 0 | 0.2 | 0 | 0 | 0.1 | 0 | 0 | 0 | 0.1 | 0.5 |
| 14 | 0 | 0 | 0.1 | 0.1 | 0 | 0 | 0 | 0 | 0 | 0 | 0 | 0 | 0 | 0.1 | 0 |
| 16 | 0.2 | 0.1 | 0 | 0 | 0 | 0.2 | 0 | 0 | 0 | 0 | 0.1 | 0.2 | 0.1 | 0.1 | 0.1 |
| 17 | 0.1 | 0 | 0.2 | 0 | 0.1 | 0.4 | 0.2 | 0.1 | 0.1 | 0.1 | 0 | 0 | 0 | 0 | 0.1 |
| 18 | 0.1 | 0 | 0.1 | 0 | 0.1 | 0.3 | 0 | 0.1 | 0 | 0.2 | 0 | 0 | 0.2 | 0 | 0 |
| 19 | 0.2 | 0.2 | 0.2 | 0 | 0 | 0 | 0 | 0.1 | 0.1 | 0 | 0.3 | 0.3 | 0 | 0 | 0.1 |
| 20 | 0.1 | 0 | 0 | 0 | 0 | 0 | 0 | 0.1 | 0.1 | 0.1 | 0.1 | 0 | 0 | 0.1 | 0 |
| 21 | 0.1 | 0.1 | 0 | 0.2 | 0.1 | 0.2 | 0.1 | 0 | 0 | 0.1 | 0.4 | 0.1 | 0 | 0.1 | 0 |

*Only subjects showing at least one positive response are included. Data are expressed as percentages of IFNγ secreting T cells/10^5^ PBMC.

| **Supplementary Table 6. Frequencies of IFNγ secreting CD8+ T cells upon peptide stimulation at T1 analyzed by CIS.** | | | | | | | | | | | | | | | |
| --- | --- | --- | --- | --- | --- | --- | --- | --- | --- | --- | --- | --- | --- | --- | --- |
| **Subject n.*** | **Stimulatory peptide(s)** | | | | | | | | | | | | | | |
|  | **A**  **0.1μg/ml** | **A**  **1μg/ml** | **A**  **10μg/ml** | **B**  **0.1μg/ml** | **B**  **1μg/ml** | **B**  **10μg/ml** | **C**  **0.1μg/ml** | **C**  **1μg/ml** | **C**  **10μg/ml** | **D**  **0.1μg/ml** | **D**  **1μg/ml** | **D**  **10μg/ml** | **ABCD 0.1μg/ml** | **ABCD 1μg/ml** | **ABCD 10μg/ml** |
| 1 | 0.2 | 0.4 | 0 | 0.2 | 0 | 0.2 | 0.4 | 0.1 | 2.2 | 0 | 0.1 | 0.2 | 1.2 | 1.2 | 0 |
| 2 | 0 | 0 | 0 | 0.2 | 0 | 0.8 | 0 | 0 | 0 | 0.1 | 0 | 0 | 0.2 | 0 | 0 |
| 3 | 0 | 0 | 0.1 | 0 | 0.4 | 0 | 0 | 0 | 0.5 | 0 | 0 | 0 | 0 | 0 | 0 |
| 4 | 1.4 | 0.2 | 0 | 0 | 0.5 | 0 | 0 | 0.3 | 0 | 0 | 0.1 | 0 | 0.2 | 0 | 1.5 |
| 5 | 0 | 0.1 | 0 | 0.3 | 0 | 0.3 | 0.1 | 0 | 0 | 0.1 | 0.2 | 0 | 2.3 | 0.4 | 0 |
| 6 | 0.2 | 0 | 0 | 0.1 | 0 | 0.4 | 0.1 | 0 | 0 | 0.3 | 0.1 | 0 | 1.1 | 0.3 | 0.3 |
| 7 | 0.2 | 0.5 | 0.1 | 0.7 | 0 | 0 | 0 | 0 | 0 | 0 | 0 | 0 | 0 | 0 | 0 |
| 8 | 0 | 0.1 | 0 | 0 | 0.2 | 0 | 0 | 0 | 0 | 0.1 | 0 | 0 | 0 | 0 | 0 |
| 9 | 0.7 | 0.2 | 0.5 | 0.1 | 0 | 0.3 | 0 | 0.1 | 0 | 0.4 | 1.2 | 0.5 | 0 | 0 | 0 |
| 10 | 0 | 2.4 | 0 | 0 | 0 | 3.1 | 0 | 0 | 0 | 0 | 0.8 | 0.7 | 0 | 0 | 0 |
| 11 | 0.6 | 0.3 | 0 | 0 | 0 | 0 | 0 | 0.3 | 0.1 | 0 | 0 | 0.2 | 0.1 | 0 | 0 |
| 12 | 0.3 | 0.1 | 0 | 0 | 0 | 0 | 0 | 0 | 0 | 0 | 0 | 0 | 0 | 0 | 0 |
| 13 | 0 | 0 | 0 | 0 | 0.2 | 0 | 0 | 0 | 0 | 0 | 0 | 0 | 0 | 0 | 0.3 |
| 14 | 0 | 0 | 0,6 | 0 | 0 | 0 | 0.1 | 0 | 0 | 0 | 0 | 0 | 0 | 0.2 | 0.1 |
| 16 | 0 | 0.3 | 0 | 0.3 | 0.2 | 0 | 0.7 | 0.1 | 0.1 | 1.4 | 0.3 | 0.5 | 0.2 | 0 | 0 |
| 17 | 0.1 | 1 | 0.2 | 0 | 0.2 | 0.1 | 0.3 | 0 | 0 | 0.1 | 0 | 0 | 0.9 | 1.3 | 0.1 |
| 18 | 0.2 | 0 | 1 | 0.7 | 0 | 0 | 0 | 0 | 0.1 | 1.3 | 0.2 | 0.2 | 0 | 0 | 0 |
| 19 | 0.5 | 0 | 0.7 | 0 | 0.6 | 0.4 | 0.7 | 0.6 | 0 | 0.2 | 0 | 1 | 0 | 0.1 | 0 |
| 20 | 0.3 | 0.2 | 0.1 | 0.2 | 0.1 | 0 | 0.1 | 0 | 0 | 0 | 0 | 0 | 0.2 | 0 | 0 |
| 21 | 0.5 | 0 | 0 | 0.3 | 0 | 0 | 0.6 | 0 | 0 | 0.6 | 0.3 | 0.1 | 0 | 0.2 | 0 |

*Only subjects showing at least one positive response are included. Data are expressed as percentages of IFNγ secreting T cells/10^5^ PBMC.

| **Supplementary Table 7. Total number of responses to peptide stimulation at T0 analyzed by either ELISPOT or CIS** | | | | | |
| --- | --- | --- | --- | --- | --- |
|  | **Stimulatory peptide(s)** | | | | |
|  | **A** | **B** | **C** | **D** | **ABCD** |
| **Total n° of responses by ELISPOT** | 6 | 17 | 16 | 8 | 21 |
| **Total n° of responses by CIS (CD4+CD8= total)** | 34+35= 69 | 36+37= 73 | 34+40= 74 | 40+39= 79 | 34+36= 70 |

| **Supplementary Table 8. Total number of responses to peptide stimulation at T1 analyzed by either ELISPOT or CIS** | | | | | |
| --- | --- | --- | --- | --- | --- |
|  | **Stimulatory peptide(s)** | | | | |
|  | **A** | **B** | **C** | **D** | **ABCD** |
| **Total n° of responses by ELISPOT** | 25 | 17 | 23 | 22 | 19 |
| **Total n° of responses by CIS (CD4+CD8= total)** | 31+32= 63 | 20+26= 46 | 16+20= 36 | 22+27= 49 | 22+21= 43 |

| **Supplementary Table 9. Total number of responders to peptide stimulation at T0 analyzed by either ELISPOT or CIS** | | | | | |
| --- | --- | --- | --- | --- | --- |
|  | **Stimulatory peptide(s)** | | | | |
|  | **A** | **B** | **C** | **D** | **ABCD** |
| **Total n° of responders by ELISPOT** | 4 | 11 | 10 | 6 | 10 |
| **Total n° of responders by CIS** | 18 | 19 | 17 | 18 | 17 |

| **Supplementary Table 10. Total number of responders to peptide stimulation at T1 analyzed by either ELISPOT or CIS** | | | | | |
| --- | --- | --- | --- | --- | --- |
|  | **Stimulatory peptide(s)** | | | | |
|  | **A** | **B** | **C** | **D** | **ABCD** |
| **Total n° of responders by ELISPOT** | 13 | 9 | 14 | 12 | 11 |
| **Total n° of responders by CIS** | 20 | 18 | 16 | 17 | 15 |

| **Supplementary Table 11. HLA class I haplotype of donors N. 2, 13, 15, 17.** | |
| --- | --- |
| **Subject N.** | **HLA class I haplotype** |
| 2 | A03, A24; B49, B50; BW4, BW6 |
| 13 | A01, A02; B08, B51; BW4, BW6 |
| 15 | A01, A02; B08; BW6 |
| 17 | A*03, A*26; B*35, B*51; BW4, BW6 |
